# Exploration of the association between quality of life, assessed by the EQ-5D and ICECAP-O, and falls risk, cognitive function and daily function, in older adults with mobility impairments

**DOI:** 10.1186/1471-2318-12-65

**Published:** 2012-10-24

**Authors:** Jennifer C Davis, Stirling Bryan, Rob McLeod, Jessica Rogers, Karim Khan, Teresa Liu-Ambrose

**Affiliations:** 1 Centre for Clinical Epidemiology and Evaluation, Vancouver Coastal Health Research Institute, The University of British Columbia, Research Pavilion, 7th floor, 828 West 10th Avenue, V5Z 1M9, Vancouver, BC, Canada; 2 Department of Physical Therapy, University of British Columbia, 212-2177 Wesbrook Mall, V6T 1Z3, Vancouver, BC, Canada; 3 School of Population & Public Health, University of British Columbia, 2206 East Mall Vancouver, BC V6T 1Z3, Vancouver, BC, Canada; 4 Centre for Hip Health and Mobility, Robert HN Ho Research Centre, 2635 Laurel Street, V5Z 1M9, Vancouver, BC, Canada

**Keywords:** ICECAP-O, EQ-5D, Construct validity, Older adults, Falls

## Abstract

**Background:**

Our research sought to understand how falls risk, cognitive function, and daily function are associated with health related quality of life (using the EuroQol-5D) and quality of life (using the ICECAP-O) among older adults with mobility impairments.

**Methods:**

The EQ-5D and ICECAP-O were administered at 12 months post first clinic attendance at the Vancouver Falls Prevention Clinic. We report descriptive statistics for all baseline characteristics collected at first clinic visit and primary outcomes of interest. Using multivariate stepwise linear regression, we assessed the construct validity of the EQ-5D and ICECAP-O using three dependent measures that are recognized indicators of “impaired mobility” – physiological falls risk, general balance and mobility, and cognitive status among older adults.

**Results:**

We report data on 215 seniors who attended the Vancouver Falls Prevention Clinic and received their first clinic assessment. Patients had a mean age of 79.3 (6.2) years. After accounting for known covariates (i.e., age and sex), the ICECAP-O domains explained a greater amount of variation in each of the three dependent measures compared with the EQ-5D domains.

**Conclusion:**

Both the EQ-5D and ICECAP-O demonstrate associations with falls risk and general balance and mobility; however, only the ICECAP-O was associated with cognitive status among older adults with mobility impairments.

**Trial registration:**

ClinicalTrials.gov Identifier: NCT01022866

## Background

Health related quality of life (HRQoL) is an important construct for healthy aging; it describes an individual’s overall health status [1]. Previous studies have demonstrated significant associations between self-efficacy, mobility, cognition and HRQoL [2]. Specifically, HRQoL is highly associated with mobility impairments and cognitive status in older adults [3-5]. Critically, functional abilities such as walking are associated with changes in both physical and mental HRQoL [6].

Impaired mobility is also significantly associated with quality of life (QoL) [7] – a construct that is distinct from HRQoL in that it captures gains or losses to an individual’s QoL beyond considering health alone. Specifically, older adults with impaired mobility experience a multitude of consequences beyond health including: loss of independence and social isolation [8]. Thus, current evidence strongly suggests older adults with impaired mobility are at significant risk for decline in both HRQoL and QoL [9]. Further, QoL is associated with cognitive status in older adults [10].

One key question remains unanswered: Are we adequately assessing HRQoL and QoL among older adults with mobility impairments? To address this question, we first need to ascertain the association between falls risk, cognitive function, and general balance and mobility, and health-related quality of life and quality of life among older adults with mobility impairments.

Two examples of feasible measures as relevant tools for this population of older adults that evaluate HRQoL and QoL, respectively, include the EQ-5D and the ICECAP-0 [11,12].

The most widely used utility-based measure of HRQoL is the EQ-5D [12-14]. The association of falls risk, cognitive function and general balance and mobility to HRQoL as assessed using the EQ-5D among older adults with mobility impairments remains unknown. The EQ-5D assesses an individual’s HRQOL according to the following attributes: mobility, self-care, usual activities, pain, anxiety and depression [12]. The EQ-5D yields a single summary score, anchored at zero (equivalent to death) and 1.0 (‘full health’). Values of less than zero define health states worse than death.

The Index of Capability for older adults (ICECAP-O) is a relatively new measure developed to provide a broader assessment of QoL among older adults [11,15]. The ICECAP-O measure covers attributes of capability found to be important determinants of QoL among older adults in the UK [11,15] – its descriptive system results from an extensive qualitative investigation [15]. The measure comprises five attributes:

•Attachment (love and friendship)

•Security (thinking about the future without concern)

•Role (doing things that make you feel valued)

•Enjoyment (enjoyment and pleasure)

•Control (independence)

The value system for the ICECAP-O provides a single summary score, anchored at zero (‘no capability’) and 1.0 (‘full capability’), for each state described in terms of the five attributes.

There remains a gap in our current knowledge regarding the understanding of the association of falls risk, cognitive function and general balance and mobility with HRQoL (assessed using the EQ-5D) and quality of life (assessed using the ICECAP-O) among older adults with mobility impairments. Hence, we aim to examine the association of the EQ-5D with the ICECAP-O with valid and reliable measures of physiological falls risk, general balance and mobility, and cognitive status among older adults with mobility impairments.

## Method

### Study design

We conducted a cross-sectional analysis of participants visiting the Vancouver Falls Prevention Clinic ( http://www.fallsclinic.com) due to a fall from January 2009 through May 2011.

### Participants

The sample consisted of women and men referred by their general practitioner or emergency department physician to the Vancouver Falls Prevention Clinic. From January 2009 through January 2011, all patients presenting to the Vancouver Falls Prevention Clinic were invited to participate. Community dwelling women and men who lived in the lower mainland region of British Columbia were eligible for study entry if they:

•were adults ≥ 70 years referred by a medical professional to the Vancouver Falls Prevention Clinic as a result of seeking medical attention for a non-syncopal fall in the previous 12 months;

•understood, spoke, and read English proficiently;

•had a Mini-Mental State Examination (MMSE) [16] score ≥ 24/30;

•had a Physiological Profile Assessment (PPA) [17] score of at least 1.0 SD above age-normative value;

•were expected to live ≥ 12 months;

•were able to walk 3 metres; and

•were able to provide written informed consent

We excluded those with a neurodegenerative disease (e.g., Parkinson’s disease) or dementia, patients who recently had a stroke, those with clinically significant peripheral neuropathy or severe musculoskeletal or joint disease, and anyone with a history indicative of carotid sinus sensitivity (i.e., syncopal falls).

Ethical approval was obtained from the Vancouver Coastal Health Research Institute and the University of British Columbia’s Clinical Research Ethics Board. All participants provided written informed consent.

### Measures

The data presented in this paper include baseline characteristics collected at participants’ first clinic assessment. The ICECAP-O and EQ-5D, were collected once at 12 months post first clinic assessment.

### Clinical indicators of falls risk, mobility, and cognitive status

As part of the clinical assessment in the Falls Prevention Clinic visit, a comprehensive set of questionnaires and clinical tests were administered. From these, we selected three measures as key indicators of “impaired mobility” -- physiological falls risk, general balance and mobility, and cognitive status among older adults. These measures were chosen based on the rationale that physiological functions such as vision, proprioception, strength, reaction time, and postural stability are significantly associated with falls risk [18-20]. Impaired mobility, balance, and cognitive function are also key risk factors for falls.

For physiological falls risk, we used the PPA © [17] (Prince of Wales Medical Research Institute, AUS). The PPA is used and recognized internationally in by both clinicians and researchers in falls prevention. It is a valid and reliable tool for falls risk assessment and this measure has a 75% predictive accuracy for falls in older people [17]. The PPA computes a falls risk score for each individual based on the individual’s performance of five physiological domains (postural sway, hand reaction time, quadriceps strength, proprioception, and edge contrast sensitivity). A PPA z-score of 0–1 indicates mild risk, 1–2 indicates moderate risk, 2–3 indicates high risk, and 3 and above indicates marked risk [19].

We assessed general balance and mobility using the Short Physical Performance Battery (SPPB) [21]. For the SPPB, participants were assessed on performances of standing balance, walking, and sit-to-stand. Each component is rated out of four points, for a maximum of 12 points. Poor performance, indicated by a score of 9 or less, on this scale predicts subsequent disability [21].

For global cognitive function, we used the MMSE [16]. A score 24/30 or greater indicates intact global cognitive function.

For assessing activities of daily living, we used the **Instrumental Activities of Daily Living (IADLs) Scale.** Participants completed the Lawton and Brody [22] Instrumental Activities of Daily Living Scale to screen for impaired IADLs. This scale subjectively assesses ability to telephone, shop, prepare food, housekeep, do laundry, handle finances, be responsible for taking medication and determining mode of transportation.

### Health related quality of life

#### EQ-5D

The EQ-5D is a short five-item multiple choice questionnaire that measures an individual’s HRQoL and health status according to the following five domains: mobility, self-care, usual activities, pain and anxiety/depression [12]. Each domain has three possible options that either indicates no problems, some problems or severe problems. The EQ-5D health state utility values (HSUVs) at each time point are bounded from −0.54 to 1.00 where a score of less than zero is indicative of a health state worse than death. The HSUVs represent values that individuals within society assign – values for specific health states such as having rheumatoid arthritis relative to perfect health – these are UK societal values for given health states.

#### Qualify of life

##### ICECAP-O

We assessed QoL using the ICECAP-O [11,15]. The ICECAP-O is a short five-item multiple choice questionnaire that measures an individual’s overall QoL according to the following five attributes: attachment (love and friendship), security (thinking about the future without concern), role (doing things that make you feel valued), enjoyment (enjoyment and pleasure) and control (independence). Each domain has four possible options. The ICECAP-O can be used to calculate a global score on a zero to one scale where zero represents no capability and one represents full capability. The ICECAP-O can also be converted to a utility scale to provide further comparability with other generic preference based instruments [23].

#### Statistical analysis

Descriptive statistics were used to characterize the study sample.

#### Bivariate analyses

To measure the association between the EQ-5D and the ICECAP-O global scores with the PPA, SPPB, and MMSE we estimated the Pearson correlation coefficients. We used Spearman correlation coefficients for the specific domains of the EQ-5D (mobility, self-care, usual activities, pain and depression) & ICECAP-O (attachment, security, role, enjoyment and control) with the PPA, SPPB, MMSE and IADLs.

#### Multivariate analyses

We conducted three separate stepwise multivariate linear regression models with valid and reliable measures of falls risk, general balance and mobility and cognitive status as the dependent variables. An alpha level of 0.1 was used for the stepwise selection approach. All regression models were bootstrapped with 1000 replications to determine the consistency of each of the three final multivariate models. Within each of these three models, we adjusted for known covariates including age and sex. Our key independent variables of interest were the EQ-5D domains (mobility, self-care, usual activities, pain, anxiety/depression) and ICECAP-O domains (attachment, security, role, enjoyment and control). For all domains of the EQ-5D and the ICECAP-O, level 1 was used as the reference category. Level 1 for both instruments indicates no problems with the domain of interest. All statistical analyses were performed using STATA Version11.0.

## Results

### Participants

Descriptive statistics for all baseline clinical variables and primary outcomes are reported in Table 1. The mean (SD) age of the cohort was 78.7 (6.2) years (n=215). On average, the cohort was at moderate risk for falling as indicated by a PPA score of 1.7 (1.2). The mean SPPB score was 7.5 (3.7) indicating poor balance and mobility and subsequent risk for disability. The majority of participants were cognitively intact as indicated by a MMSE score of 26.9 (3.4). The mean IADL score was 7.0 (1.6) with 42% of participants indicating IADL impairment at baseline. The mean EQ-5D global score was 0.701 (9.291) and the mean ICECAP-O score was 0.815 (0.177).

**Table 1:** Characteristics of the Falls Prevention Clinic cohort (N=215)

**Variable**	**Mean (SD) or Median (IQR) Or Number (%)**
Age (years)	79.3 (6.2)
Sex (female)	154 (71.6)
Body Mass Index (kg/m^2^)	27.1 (5.9)
Instrumental Activities of Daily Living (max 8 points)	7.0 (1.6)
Physiological Profile Assessment	1.7 (1.2)
Short Performance Physical Battery (max 12 points)	7.5 (3.7)
Mini Mental State Examination (max 30 points)	26.9 (3.4)
EQ-5D Global Score (0–1 scale)	0.701 (0.291)
ICECAP-O Global Score (0–1 scale)	0.815 (0.177)

### Bivariate analyses

Table 2 details the results of the bivariate analyses. The global scores of EQ-5D and ICECAP-O were significantly correlated (r^2^ = 0.474; p<0.01).

**Table 2:** Correlation coefficient matrix summary for measures of fall risk, mobility, cognitive status and activities of daily living versus health related quality of life and quality of life domains

	**Physiological profile assessment**	**Short physical performance battery**	**Mini-mental state examination**	**Instrumental activities of daily living**
EQ-5D Global Score	0.013	0.060	−0.068	0.028
Mobility	−0.078	−0.177*	0.043	0.024
Self-Care	0.071	−0.076	−0.007	−0.238*
Usual activities	−0.008	−0.149*	−0.046	−0.101
Pain	−0.066	−0.124	0.124	0.006
Depression	−0.086	−0.009	0.110	0.072
ICECAP-O Global Score	−0.042	0.067	0.006	0.097
Attachment	0.078	0.026	−0.071	0.074
Security	0.081	0.115	−0.169*	0.033
Role	−0.021	0.175*	0.008	−0.230*
Enjoyment	−0.029	0.097	−0.023	−0.167*
Control	−0.192*	0.299*	0.107	−0.392*

*p<0.05.

None of the domains of the EQ-5D were significantly correlated with the PPA or MMSE (p>0.05). The EQ-5D domain of mobility (r^2^ = −0.177, p<0.05) was significantly correlated with the SPPB. The EQ-5D domain of self-care (r^2^ = −0.238, p<0.05) was significantly correlated with IADLs. For the ICECAP-O, the domain ‘control’ was significantly associated with the PPA (r^2^ = −0.192, p<0.05), the domains ‘role’ and ‘control’ were significantly associated with the SPPB (r^2^ = 0.175, p<0.05; r^2^ = 0.299, p<0.05), the domain ‘security’ was significantly associated with the MMSE (r^2^ = −0.169, p<0.05) and the domains of role (r^2^ = −0.230, p<0.05), enjoyment (r^2^ = −0.167, p<0.05) and control (r^2^ = −0.392, p<0.05) were significant associated with IADLs.

### Multivariate analyses

Table 3 details the results of the multivariate analyses.

**Table 3:** Multivariate linear regression summary for measures of fall risk, mobility, cognitive status and activities of daily living versus health related quality of life and quality of life

**Independent variables**	**Physiological profile assessment**	
	**Unstandardized ß (Standard Error)**	**P-value**
Model 1	R^2^ 19.70	0.0001
Age	0.024 (0.014)	0.092
Sex (Reference = Female)	-0.129 (0.190)	0.498
Mobility_2 (Reference=1)	-0.317 (0.184)	0.087
Usual Activities_3 (Reference=1)	0.929 (0.347)	0.008*
Role_4 (Reference=1)	3.181 (1.085)	0.004**
Enjoyment_4 (Reference=1)	-3.138 (1.192)	0.009*
Control_2 (Reference=1)	0.566 (0.193)	0.004*
Control_3 (Reference=1)	0.652 (0.261)	0.014*
	Short performance physical battery	
Model 2	R^2^ 27.86	0.000**
Age	-0.148 (0.044)	0.001**
Sex (Reference=Female)	-0.391 (0.618)	0.528
Self care_3 (Reference=1)	15.098 (2.099)	0.000**
Security_3 (Reference=1)	-1.284 (0.646)	0.049*
Security_4 (Reference=1)	-2.395 (1.011)	0.019*
Control_4 (Reference=1)	-3.629 (1.802)	0.046*
	Mini-mental state examination	
Model 3	R^2^ 14.33	0.0008**
Age	-0.073 (0.034)	0.033*
Sex (Reference=Female)	0.095 (0.483)	0.845
Security_2 (Reference=1)	0.970 (0.484)	0.047*
Security_3 (Reference=1)	1.444 (0.570)	0.012*
Security_4 (Reference=1)	2.340 (0.920)	0.012*
Role_4 (Reference=1)	-3.124 (1.204)	0.010*
Control_3 (Reference=1)	-1.579 (0.551)	0.005*
	Instrumental activities of daily living	
Model 4	R^2^ 19.48	0.0001**
Age	-0.022 (0.017)	0.202
Sex (Reference=Female)	0.277 (0.244)	0.258
Self-care_2 (Reference=1)	-0.673 (0.283)	0.019*
Pain_2 (Reference=1)	0.413 (0.217)	0.059
Control_3 (Reference=1)	-0.822 (0.299)	0.007*
Control_4 (Reference=1)	-2.294 (0.710)	0.001*
Security_3 (Reference=1)	0.492 (0.276)	0.076
Security_4 (Reference=1)	1.042 (0.436)	0.018*
Role_3 (Reference=1)	-0.547 (2.61)	0.038*

* p < 0.05.

** p < 0.001.

### Physiological falls risk

After accounting for known covariates (i.e., age and sex), we found that the ICECAP-O domains of role (level 4), enjoyment (level 4) and control (levels 2 and 3) explained a statistically significant amount of variation in the PPA score (p < 0.05). Mobility (level 2), a domain of the EQ-5D explained a non-significant degree of variation in the PPA score. Depression (level 2) and Usual Activities (level 3) of the EQ-5D explained a statistically significant amount of variation in the PPA score (p < 0.05).

### General balance and mobility

After accounting for known covariates, we found that the ICECAP-O domains of security (levels 3 and 4) and control (level 4) explained a statistically significant amount of variation in the SPPB score (p < 0.05). Self-care (level 3), a domain of the EQ-5D explained a significant degree of variation in the SPPB score (p < 0.05).

### Cognitive status

After accounting for known covariates, we found that the ICECAP-O domains of control (level 3), security (levels 2, 3 and 4) and role (level 4) explained a statistically significant amount of variation in the MMSE score (p < 0.05). None of the domains of the EQ-5D explained a significant degree of variation in the MMSE score.

### Instrumental activities of daily living

After accounting for known covariates (i.e., age and sex), we found that the ICECAP-O domains of role (level 3), security (levels 4) and control (levels 3 and 4) explained a statistically significant amount of variation in IADLs (p < 0.05). Pain (level 2), a domain of the EQ-5D explained a non-significant degree of variation in IADLs. Self-care (level 2), a domain of the EQ-5D explained a statistically significant amount of variation in IADLs (p < 0.05).

## Discussion and conclusions

### Principal findings

Our data suggest that the EQ-5D and ICECAP-O are significantly correlated. Of note, a greater number of domains of the ICECAP-O compared with the EQ-5D explain significant variation in the PPA, SPPB, MMSE and IADLs. Using three key indicators of “impaired mobility”, we demonstrated several distinct differences between select domains of the EQ-5D and the ICECAP-O. Specifically, role, enjoyment and control (ICECAP-O domains) explained a statistically significant amount of variation in falls risk. Depression and usual activities (EQ-5D domains) explained a statistically significant amount of variation in falls risk. Security and control (ICECAP-O domains) explained a statistically significant amount of variation in general balance and mobility. Self-care (EQ-5D domain) explained a significant degree of variation in general balance and mobility. Control, security and role (ICECAP-O domains) explained a statistically significant amount of variation in the cognitive status and in IADLs; whereas, similar findings were not observed for any of the EQ-5D domains. Hence, our findings suggest that among older adults with impaired mobility, both instruments provide valuable yet unique information.

### Strengths and weaknesses of this study

A key strength of this study is that it compares the construct validity of the ICECAP-O and the EQ-5D in a specific population of older adults with impaired mobility. Given that the ICECAP-O is a relatively new instrument, this study provides a population specific recommendation for use of the ICECAP-O in select samples. This comparison is also useful given that the EQ-5D is a widely used instrument. It also provides a benchmark from which future studies can compare the construct validity of these and other widely used instruments such as the Short Form -6D [24] and Health Utilities Index [25].

We recognize that the analyses in this paper are cross-sectional and are therefore unable to infer causation. Further, the small sample size may have limited our ability to detect a statistically significant difference. To address this limitation, we conducted 1000 bootstrap replications of the three stepwise linear regression models. Lastly, our study is population specific to older adults with mobility impairment and did not include individuals with significant cognitive impairment. Thus, we are not able to generalize our findings to such broader populations.

### Comparison with other research

To our knowledge, only one study has examined associations of the ICECAP-O in a population of older adults [15]. This study demonstrated the first evidence of associations between the capability measure and measures of functioning [15]. Although a few studies have examined the validity of the EQ-5D in a general population of adults including older adults [26,27], to date, there is no established validation work for the EQ-5D among older adults with mobility impairments.

### Implications for research and practice

The study of older adults with mobility impairments is essential because injuries in this population are associated with increased morbidity, decreased functioning and increased healthcare resource utilization [20,28-30]. Our findings are the first to highlight that both the EQ-5D and the ICECAP-O are associated with reliable and valid markers of falls risk and general balance and mobility among older adults with mobility impairments; however, the ICECAP-O captures key indicators of impaired mobility better. Further, the ICECAP-O also captures key indicators of cognitive function better. As such, this study provides a platform for recommending the ICECAP-O to assess QoL among older adults with mobility impairments.

## Conclusion

Our study suggests that both the EQ-5D and ICECAP-O demonstrate associations with falls risk and general balance and mobility; however, only the ICECAP-O was associated with cognitive status among older adults with mobility impairments.

## Competing interests

The authors declare that they have no competing interests.

## Authors’ contributions

JCD was principal investigator for the evaluation of the association between quality of life, assessed by the EQ-5D and ICECAP-O, and falls risk, cognitive function and daily function and was responsible for design, data analysis and interpretation, and writing of manuscript. TLA was principal investigator for the study and was responsible for study concept and design, acquisition of data, data analysis and interpretation, writing and reviewing of the manuscript. RM and JR were responsible for data collection and critical review of manuscript. KK was responsible for study concept and critical review of the manuscript. SB was responsible for data analysis and interpretation, writing and reviewing of the manuscript. All authors read and approved the final manuscript.

## Pre-publication history

The pre-publication history for this paper can be accessed here:

http://www.biomedcentral.com/1471-2318/12/65/prepub
